# Hydrophobia

**Published:** 1880-11

**Authors:** 


### HYDROPHOBIA.

**A** member of the Society of Friends sends a recipe for the cure of this terrible affliction, as coming from one whom he knows to be a reliable man, but it is not here given, because it would mislead. The facts about Hydrophobia are these. The great John Hunter estimated that not more than one person out of twenty-one bitten by a rabid animal became Hydrophobic. In a case lately reported, out of a large number of animals bitten by a mad dog, only one died, or had any of the symptoms of the dreaded malady. All the so called "cures" which have come to our notice, are things which have been done immediately after the bite, and because the bitten person gave no indication of being hydrophobic, that thing is heralded as a cure. It is in this manner the "Mad-stone," so implicitly believed in by some, has gained its celebrity ; it is well known that it has signally failed of any virtue whatever in some cases. By teaching the people that this, that and the other is a cure for the malady, they may rely on a broken reed, and, while so relying, may loose a life which might have been saved by the prompt application of the surgeon's knife or the cautery : we would say to any one bitten by a rabid animal, known to be so, have it cut out or burned with caustic by the nearest physician at the earlist possible moment. Persons have suffered for years the horrible mental torture of apprehension by having been bitten by an animal only supposed to be mad, and which by having been killed at once cannot be proven to have been mad ; on this account it is best when a person is bitten by an animal to cage it, if possible, instead of killing it, for many a time it has happened that the supposed madness is only terror which would subside in a few hours by kind treatment or rest and sleep.

The saliva of a mad dog has no effect whatever on a broken skin. The most indisputable signs that a dog is mad are, 1st. He is sullen. 2d. Scratches his ear violently. 3d. Paws the corners of his mouth, *without* its being permanently open.

As to the article so highly recommended by our correspondent, which was given to animals and men, actually bitten by mad dogs or *supposed* to have been so bitten, not one of them was actually attacked with the first symptom ; the evidence is entirely negative, they were bitten, took the remedy and were not attacked, but to say they were cured and that too when not a single symptom of it was observed is going entirely too far ; for in twenty of John Hunter's cases doing little or nothing after the bite, no harm came of it. We will gladly publish any remedy which arrests the actual throes of this terrible infliction.

